# Dietary Bile Salt Types Influence the Composition of Biliary Bile Acids and Gut Microbiota in Grass Carp

**DOI:** 10.3389/fmicb.2018.02209

**Published:** 2018-09-18

**Authors:** Fan Xiong, Shan-Gong Wu, Jing Zhang, Ivan Jakovlić, Wen-Xiang Li, Hong Zou, Ming Li, Gui-Tang Wang

**Affiliations:** ^1^Key Laboratory of Aquaculture Disease Control, Ministry of Agriculture, Institute of Hydrobiology, Chinese Academy of Sciences, Wuhan, China; ^2^State Key Laboratory of Freshwater Ecology and Biotechnology, Institute of Hydrobiology, Chinese Academy of Sciences, Wuhan, China; ^3^University of Chinese Academy of Sciences, Beijing, China; ^4^Bio-Transduction Lab, Wuhan Institute of Biotechnology, Wuhan, China

**Keywords:** bile acid, gut microbiota, lipid metabolism, freshwater fish, *in vivo*

## Abstract

Lipid metabolism can influence host’s health. There is increasing evidence for interplay between two key regulating factors in lipid metabolism: bile acids (BAs) and gut microbiota. However, very little is known about how types of different diet-supplemented bile salts (BS) influence this interaction *in vivo*. We sought to explore these relationships using grass carp (*Ctenopharyngodon idellus*), which often suffers functional disorder of liver and gallbladder. We studied fluctuations of BAs in the gall and changes of microbial communities in the gut in response to seven different diets: five different BS, chelating BS agent, and control. The BS comprised two primary BS [sodium taurochololate (TCAS) and sodium taurochenodeoxycholate (TCDCAS)], sodium tauroursodeoxycholate (TUDCAS), and two secondary BS [sodium taurodeoxycholate (TDCAS) and sodium taurolithocholate (TLCAS)]. Supplementation of primary BS caused a more significant fluctuation of biliary BAs than secondary BS, and TCAS caused a more prominent increase than TCDCAS and TUDCAS. For the gut microbiota, primary BS tended to increase their diversity and induce community succession, secondary BS resulted in a higher firmicutes/bacteroidetes ratio, while TUDCAS had no significant effects. Changes of the gut microbiota triggered by different types of BS caused alteration in BAs biotransformation. Two-obesity-associated families, Lachnospiraceae and Ruminococcaceae were positively correlated with biliary cholic acid (CA), taurochenodeoxycholic acid (TCDCA), and deoxycholic acid (DCA). As both primary and secondary BS resulted in increased synthesis of toxic secondary Bas by the gut microbiota, future studies should pay closer attention to gut microbiota when considering BA treatment.

## Introduction

Lipid metabolism has a role in host’s health. Disorders of lipid metabolism lead to serious, sometimes life-threatening, health problems, including obesity, cardiovascular, and hepatobiliary diseases (Lee et al., 2003; Reddy and Rao, 2006). High-fat diets can cause these metabolic disorders both directly and indirectly via changes in gut microbiota (Tremaroli and Bäckhed, 2012; Daniel et al., 2014). Although the latter issue has recently received ample scientific attention, the intricacies of the complex interplay among the high-fat diets, gut microbiota, and metabolic diseases remain poorly understood. As bile acids (BAs) are known to play an important role in a large number of metabolic disorders (Hofmann and Hagey, 2014; Zhou and Hylemon, 2014), they have been recognized as a putatively important factor in this complex interplay (Yokota et al., 2012). They are synthetized from cholesterol in liver and secreted into the intestinal lumen as bile, where they help lipid digestion and absorption. As both BAs and gut microbiota play key roles in the regulation of host’s lipid metabolism (Nittono, 1980; Kurumiya et al., 2003; Ley et al., 2006; Claesson et al., 2012; Yoshimoto et al., 2013), there is increasing evidence for a close correlation between those two factors in mammals (Yokota et al., 2012; Ridlon et al., 2014; Liu et al., 2017). BAs can affect gut microbiota both directly, as antimicrobial agents secreted into lumen of the intestine (Kurdi et al., 2006), and indirectly, as inducers of genes encoding antimicrobial peptides and lectins *via* BA receptors (D’Aldebert et al., 2009), such as farnesoid X receptor (FXR) (Inagaki et al., 2006). In return, the gut microbiota alters the physicochemical properties of BAs by degrading bile salts (BS) and generating high-affinity ligands for BA receptors (Ridlon et al., 2014). Biotransformation of BAs by gut microbiota includes BS deconjugation, carried out by BS hydrolases (BSHs), BA oxidation, and epimerization, carried out by hydroxysteroid dehydrogenases (HSDHs), and BA 7α/β-dehydroxylation, which is involved in conversion of primary to secondary BAs (Ridlon et al., 2006). Recent studies have demonstrated that gut microbiota have important roles in adjusting the levels and profiles of BAs in various tissues (e.g., liver, kidney, plasma, and heart) (Swann et al., 2011). In this regard, by altering the composition of BAs, gut microbiota has the potential to change host’s physiology and metabolism. Primary BAs are synthesized by the liver, whereas secondary BAs are products of the metabolism of gut microbiota. Decades of research have revealed a strong connection between secondary BAs [such as deoxycholic acid (DCA) and taurolithocholic acid (TLCA)] and numerous diseases in humans, including cholesterol gallstone disease (Berr et al., 1996) and obesity-associated cancers, such as liver cancer (Yoshimoto et al., 2013). Therefore, decoding the interplay between the metabolism of BAs and the gut microbiota will be extremely important for prevention and treatment of metabolic diseases. However, despite the importance of this issue, and although different species of BAs could have completely distinct effects on host metabolism (Vallim et al., 2013), apart from two studies conducted on rats and mice with one species of BAs [cholic acid (CA)] supplemented in diet (Islam et al., 2011; Zheng et al., 2017), attempts to elucidate the effects of different dietary BAs on shaping the gut microbiota *in vivo* remain sparse.

Grass carp (*Ctenopharyngodon idellus*), the major aquacultured species globally in terms of the total production output (Faoro, 2015), often suffers functional disorder of liver and gallbladder, which is accompanied by the accumulation of lipids in liver and which can result in death (Ni and Wang, 1999). It has also been reported that different diets produce distinct BA pools and gut microbiota profiles in grass carp (Zhang et al., 2017). Herein, five different BS-supplemented diets, as well as one BA-chelating diet and one control diet (no BS added), were used to conduct the grass carp feeding trial. Fluctuations of biliary BAs and gut microbiota were determined, and interactions between biliary BAs and the composition of microbiome were assessed in detail. The objectives of this study are to broaden our understanding of the BA metabolism and its impacts on gut microbiota, as well as disclose its potential impacts on metabolic diseases in grass carp.

## Materials and Methods

### Experimental Diets

Two primary BS: sodium taurochololate (TCAS) and sodium taurochenodeoxycholate (TCDCAS); two secondary BS: sodium taurodeoxycholate (TDCAS) and sodium taurolithocholate (TLCAS); and cholestyramine (resin) were purchased from Sigma-Aldrich (St. Louis, MO). Sodium tauroursodeoxycholate (TUDCAS) was purchased from Huaaobio Company (Beijing, China). TUDCAS eschews classification in this aspect, or more precisely, its classification as primary or secondary BS is host dependant. For example, UDCA is considered to be a secondary BA in humans (Hofmann, 1999; Russell, 2003), but a primary BA in rodents (Sayin et al., 2013). As its classification in fish remains uncertain, we decided not to classify it in our experiment. Seven diets were prepared; one control group (Ctrl group; no BSs added), and six experimental diets: five BS-supplemented diets and one with supplemented (10 g/kg) BA-chelating agent – cholestyramine resin (Chol group). Each of the five BS-supplemented diets contained 0.2 mmol/kg of a different BS: TCAS (TCA fed group), TCDCAS (TCDCA fed group), TUDCAS (TUDCA fed group), TDCAS (TDCA fed group), and TLCAS (TLCA fed group). The dose of BSs used here was determined based on the fact that similar dose of a mixture of BAs can promote the growth of grass carp (Chen, 2016). All seven diets were composed of 32.7% protein, 5.6% fat, and 5.0% fiber. All diets were prepared in the Institute of Hydrobiology, Chinese Academy of Sciences, and stored at 4°C till used.

### Fish and Feeding Trial

Juvenile grass carp (*n* ≈ 600) were obtained from the Wuhu Fish Hatchery (Hubei, China). Prior to the experiment, fish were reared in 1000-L plastic tanks for 2 weeks to acclimate them to the experimental conditions. After the acclimatization period, 315 fish specimens of similar size (average weight: 40 ± 5 g) were randomly distributed into 21 aquariums (125 L of water each) at the stocking density of 15 fish per tank. Each aquarium was randomly assigned a diet, and each dietary treatment was conducted in triplicate (totally 45 fish for each diet). Fish were fed to apparent satiation three times daily (08:30, 12:30, and 17:30) for 6 weeks. During the feeding trial, fish were maintained under the natural photoperiod conditions, water temperature ranged from 22 to 23°C, pH fluctuated between 7.2 and 7.4, and water replacement rate was adjusted to keep the total ammonia nitrogen and nitrites below 0.2 and 0.005 mg/L, respectively. At the end of the feeding trial, three fish specimens from each diet replicate (adding up to nine specimens per each treatment) were anesthetized in diluted tricaine methanesulfonate (MS-222, Sigma, United States) at the concentration of 100 mg/L. The fish were then euthanized, dissected, and gallbladder and hindgut content of each fish collected and stored at -80°C for the downstream use. All the samplings were done between 5 and 6 h after feeding in order to avoid the temporal variation of gut microbiota (Zhang et al., 2017). All animal-handling procedures and experiments were reviewed and approved by the ethics committee of the Institute of Hydrobiology, Chinese Academy of Sciences.

### Biliary Acids Measurement and Statistics

BAs were extracted according to the method described by Hagio et al. (2011). We used a syringe needle to puncture the gallbladder and extract bile; 20 μl (per specimen) of which was used for the extraction of BAs. We discarded seven bile samples which did not have enough bile, so only 56 bile samples (TCA fed group = 6, TCDCA fed group = 8, TUDCA fed group = 9, TLCA fed group = 8, TDCA fed group = 8, Chol = 9, and Ctrl = 8) were successfully processed. Ten BAs, including four primary: CA, taurocholic acid (TCA), chenodeoxycholic acid (CDCA), and taurochenodeoxycholic acid (TCDCA); four secondary: DCA, taurodeoxycholic acid (TDCA), lithocholic acid (LCA), and taurolithocholic acid (TLCA); as well as two non-classified BAs: ursodeoxycholic acid (UDCA) and tauroursodeoxycholic acid (TUDCA) were quantified in the bile samples of grass carp by high-performance liquid chromatography–mass spectrometry (HPLC-MS) as described before (Zhang et al., 2017). Total BAs were calculated as the sum of the concentrations of all 10 measured BAs. Pairwise comparisons of the concentration of measured BAs between any two groups were tested using *t*-tests. Permutational multivariate analysis of variance (PERMANOVA) was conducted to test for significant differences between groups in terms of overall biliary BAs composition using Bray-Curtis distance using the R Vegan package (R Core Team, 2014).

### 16S rRNA Amplicon Sequencing Library Preparation

DNA was extracted from hindgut contents of the 56 samples for which biliary BAs were measured successfully (see previous section) using QIAamp Fast DNA Stool Mini Kit (Qiagen, Germany). NanoDrop 2000 Spectrophotometer (Thermo Scientific, United States) was used to check the concentration and quality of the extracted DNA. Extracted DNA was diluted to 10 ng/μL and stored at -80°C for downstream use.

Universal primers 515F (5′-GTGYCAGCMGCCGCGGTA-3′) and 806R (5′-GGACTACHVGGGTWTCTAAT-3′), with a 12 nt unique barcode at 5′-end of 515F, were used to amplify the hypervariable V4 region of the bacterial 16S rRNA gene (Caporaso et al., 2011). Each sample was amplified in triplicate, in a 25-μL reaction mix containing 2xGoTaq Green Master Mix (Promega, United States), 1 μM of each primer and 10 ng genomic DNA. The program included 5 min at 94°C, followed by 23 cycles of 94°C for 30 s, 50°C for 30 s, and 72°C for 30 s, and finally 10 min at 72°C. PCR products were purified using the MinElute 96 UF PCR Purification Kit (Qiagen, Valencia, CA, United States). All samples were sequenced using an Illumina Hiseq platform (HiSeq Reagent Kit V2, 500 cycles) by the Novogene Biotechnology Co., Ltd (Beijing).

### Analysis of the 16S rRNA Amplicon Sequencing Data

The raw sequence data were processed using QIIME Pipeline-Version 1.8.0^1^ (Caporaso et al., 2010). Overlapping paired end reads were merged using the FLASH-1.2.8 software (Magoc and Salzberg, 2011). Only the merged sequences with high-quality reads (length >250 bp, without ambiguous bases BN, and average base quality score >30) were used for further analysis. All sequences were trimmed and assigned to each sample based on their barcodes (barcode mismatches = 0). Chimeras were removed using the Uchime algorithm (Edgar et al., 2011). Non-chimera sequences were subsampled to the same sequence depth (11,784 reads per sample) using daisychopper.pl (Gilbert et al., 2009). This subset of sequences was clustered into OTUs at 97% identity threshold using CD-HIT (Li and Godzik, 2006). Singleton sequences were filtered out. Operational taxonomic unit (OTU) identities were assigned in Greengenes database (release 13.8) (DeSantis et al., 2006) using UCLUST (Edgar, 2010). Sequences classified as unassigned and C_Chloroplast were removed. Alpha diversity (Chao1, PD_whole_tree, Shannon and Simpson index) and beta diversity (weighted UniFrac metric and Bray-Curtis distance) indices of bacterial communities were calculated.

Cluster analysis was performed on Bray–Curtis distance matrices of bacterial OTUs using an unweighted pair group mean (UPGMA) algorithm. Principal coordinate analysis (PCoA) was used to visualize similarities between groupings with weighted_unifrac distance (Navas-Molina et al., 2013). PERMANOVA analysis was performed to test for significant differences between groups in overall microbial composition with weighted_unifrac distance (Kelly et al., 2015). Pearson correlations of relative abundance at the genus level versus principal coordinate axis scores were used to identify taxa contributing to the separation of samples in the PCoA space.

Group differences in relative abundance at the genus taxonomic level were tested using the nonparametric Kruskal–Wallis test correcting for multiple comparisons with the Benjamini–Hochberg false discovery rate (FDR) procedure. Pairwise comparisons between each experimental group and Ctrl group were tested using *t*-tests.

Metagenomic content of samples was inferred from 16S rRNA gene sequence data using PICRUSt 1.0 and KEGG database, which includes 6909 bacterial genes (i.e., metagenes) annotated in the reference genomes (Langille et al., 2013). Stamp v2.1.3 (Parks et al., 2014) was used for all statistical analyses of functional profiles. Pathway differences in relative abundance associated with lipid metabolism were tested using nonparametric Kruskal–Wallis test correcting for multiple comparisons with the Benjamini–Hochberg FDR procedure. Pairwise comparisons of the abundance of candidate genes between any two groups were tested using *t*-tests.

### Correlation Analysis Between Biliary BAs and Microbial Taxa

Biliary BAs and microbiota taxa from 56 samples were analyzed and used to construct molecular ecological networks between different biliary BAs and top 50 microbial taxa at the genus level. Networks were constructed using MENA pipeline with default parameters, based on the random matrix theory (RMT) method with the RMT cutoff value set at 0.5 (Deng et al., 2012). Modules were detected using fast greedy modularity optimization. Cytoscape 3.2.1 software was used to visualize the network graphs (Shannon et al., 2003). Significance of the correlation between BAs and bacterial taxa was evaluated using the Pearson correlation coefficient (*r*).

### Accession Number

The obtained raw 16S rRNA sequences were deposited in the NCBI/EBI/DDBJ Sequence Read Archive (Bioproject: PRJNA416986).

## Results

### BA Composition in the Gallbladder of Grass Carp

**FIGURE 1:**
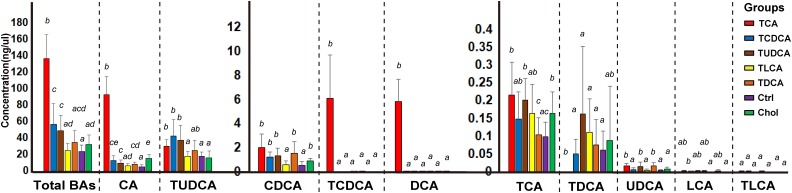
Concentrations of different biliary BAs in the galls of different groups. For the TCA, TCDCA, and Ctrl groups, *n* = 8; for the TUDCA, TLCA, TDCA, and Chol groups, *n* = 9. Different letters above bars indicate statistically significant (*P* < 0.05) in each BA (*x*-axis). Groups not sharing the same lowercase letter are significantly different (*P* < 0.05).

BAs in the gallbladder of grass carp were dominated by CA (1.0–120.1 ng/μl over groups) and TUDCA (3.6–86.1 ng/μl over all groups), followed by medium levels of CDCA (0.1–4.1 ng/μl over groups), TCDCA (0–9.3 ng/μl over groups), and DCA (0–9.1 ng/μl over groups), and low levels of TCA (0–0.4 ng/μl over groups), TDCA (0–0.6 ng/μl over groups), UDCA (0–0.03 ng/μl over groups), LCA (0–0.006 ng/μl over groups), and TLCA (0–0.0001 ng/μl over groups) (**Figure 1**). The sum of the concentrations of all measured biliary BAs (total BAs) varied greatly among the six experimental treatments. Compared with the Ctrl group, the concentration of total BAs in groups fed with primary BS (TCAS and TCDCAS) and TUDCAS showed significant increase, the groups fed with secondary BS (TDCAS and TLCAS) and chelating agent (Chol group) did not show significant increase (*P <* 0.05, **Supplementary Table S1**). The concentration of total BAs was ranked as follows: TCA fed group [5.8-fold change (fc), *P =* 0.000] > TCDCA fed group (2.4 fc, *P =* 0.008) > TUDCA fed group (2.1 fc, *P =* 0.003) > TDCA fed group (1.5 fc, *P =* 0.079) > Chol group (1.3 fc, *P =* 0.100) > TLCA fed group (1.0 fc, *P =* 0.820) > Ctrl group (1.0 fc, *P =* 1.000). In detail, TCA fed group exhibited an increased concentration of seven measured BAs, with extremely high levels of DCA (>100 fc, *P =* 0.000), TCDCA ( > 100 fc, *P =* 0.000), CA (17.8 fc, *P =* 0.000), and a moderate increase in concentrations of UDCA (4.9 fc, *P =* 0.006), CDCA (3.8 fc, *P =* 0.0196), TCA (2.2 fc, *P =* 0.024), and TUDCA (1.7 fc, *P =* 0.110); TCDCA fed group exhibited a moderate increase in concentrations of CA (2.4 fc, *P =* 0.012), TUDCA (2.4 fc, *P =* 0.010), and CDCA (2.2 fc, *P =* 0.004); TUDCA fed group showed a moderate increase in concentrations of CA (1.9 fc, *P =* 0.005), TUDCA (2.1 fc, *P =* 0.010), UDCA (4.3 fc, *P =* 0.019), CDCA (2.4 fc, *P =* 0.008), and TCA (2.1 fc, *P =* 0.001); TDCA fed group showed increased levels of CA (1.6 fc, *P =* 0.033), CDCA (2.8 fc, *P =* 0.022), and UDCA (4.6 fc, *P =* 0.011). Finally, Chol group exhibited elevated levels of CA (2.9 fc, *P =* 0.001), CDCA (1.6 fc, *P =* 0.018), and TCA (1.7 fc, *P =* 0.021).

The result of PERMANOVA test showed that biliary BAs of the TCA fed group were significantly different from all other groups (*P <* 0.05 in all cases, **Table 1**); TCDCA fed group was significantly different from all groups except TUDCA fed group; the two secondary BS groups (TDCA fed and TLCA fed groups) were not significantly different from the Ctrl group (*P =* 0.094 and *P =* 0.237, respectively). Chol group showed significant differences with all other groups (*P* < 0.011 in all cases, **Table 1**).

**Table 1:** PERMANOVA analysis of biliary BAs with Bray–Curtis distance.

	TCA	TCDCA	TUDCA	TLCA	TDCA	Ctrl	Chol
TCA		***0.0003***	***0.0002***	***0.0008***	***0.0002***	***0.0005***	***0.0004***
TCDCA	**29.63**		*0.8523*	***0.0016***	***0.0494***	***0.0015***	***0.0014***
TUDCA	**36.17**	0.16		***0.0015***	*0.0944*	***0.0009***	***0.0016***
TLCA	**40.80**	**8.86**	**7.41**		*0.2367*	*0.8838*	***0.0021***
TDCA	**32.87**	**3.39**	2.37	1.43		*0.1130*	***0.0138***
Ctrl	**77.81**	**14.06**	**8.64**	0.21	2.51		***0.0002***
Chol	**34.30**	**8.48**	**8.64**	**5.25**	**4.12**	**9.07**	


*Pseudo-F values of the PERMANOVA test are shown in regular font, P-values are italicized, and P-values < 0.05 are bolded.*

In summary, supplementation of primary BS and TUDCAS caused a more significant fluctuation of biliary BAs than secondary BS and chelating agent, and TCAS caused a more prominent increase than TCDCAS and TUDCAS; TCDCAS and TUDCAS resulted in similar effect on biliary BAs. The secondary BS (TLCAS and TDCAS) did not significantly alter biliary BAs; The chelating agent slightly promoted the concentration of biliary CA, CDCA and TCA.

### Gut Microbiota Composition and Diversity in Different Groups

**FIGURE 2:**
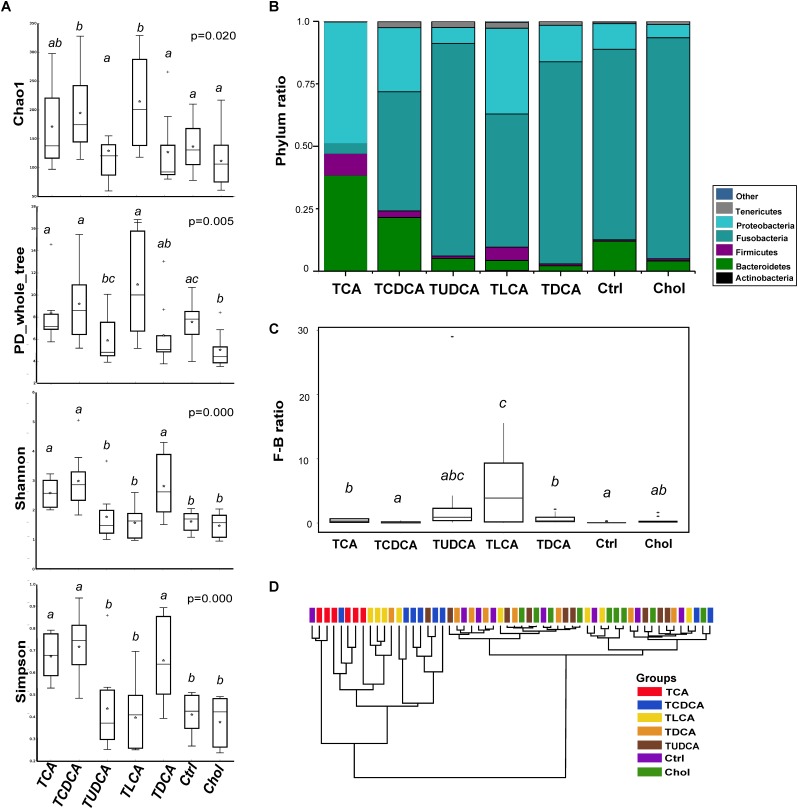
Effects of different dietary bile salts on shaping the hindgut microbiota in grass carp. **(A)** Microbial alpha diversity in each group was calculated using four different parameters: Chao1, PD_whole_tree, Shannon and Simpson. *P*-value was evaluated with Kruskal–Wallis test. **(B)** Microbial composition of each group at the phylum level. **(C)** The ratio of microbial Firmicutes/Bacteroidetes (F/B ratio) taxa in each group. **(D)** Cluster analysis performed on Bray–Curtis distance matrices of bacterial OTUs using an unweighted pair group mean (UPGMA) algorithm. Different letters above bars indicate statistically significant (*P* < 0.05) differences between groups **(A,C)**. For the TCA fed group, *n* = 6; for the TCDCA, TLCA, TDCA, and Ctrl groups, *n* = 8; for the TUDCA and Chol groups, *n* = 9.

Gut microbiota of the 56 fish samples were sequenced. After the initial quality filtering, chimera checking, and OTU picking, 624 non-singleton OTUs were identified at the 97% similarity level. At the genus level, roughly 57.4% of the total reads were annotated as *Cetobacterium*, followed by *Bacteroides* (10.2%) and *Citrobacter* (5.6%). The composition of gut microbiota varied greatly among different groups. At the phylum level (**Figure 2B**), Fusobacteria ranged from 4.1% in TCA fed group to 88.5% in Chol group. Proteobacteria, Bacteroidetes, and Firmicutes decreased from 48.5, 38.2, and 8.7% in the TCA fed group to 5.4, 4.1, and 0.8% in the Chol group, respectively. Compared with the Ctrl group, both TCA fed and TCDCA fed groups exhibited increased Bacteroidetes, Firmicutes, and Proteobacteria, and decreased Fusobacteria. Only two of these, Firmicutes and Proteobacteria, were increased in the TLCA fed group. However, in TUDCA fed group and Chol group, Proteobacteria were decreased, while Fusobacteria were increased (**Supplementary Figure S1**). The median value of Firmicutes/Bacteroidetes (F/B ratio) ranged from 0.108 in the Ctrl group to 5.594 in the TLCA fed group (**Figure 2C**). It was significantly increased in TCA fed, TLCA fed, and TDCA fed groups in comparison to the Ctrl group (*P <* 0.005 in all cases, **Supplementary Table S2**).

In comparison to the Ctrl group, Shannon and Simpson indices of two primary BS groups (TCA fed and TCDCA fed groups) and one secondary BS group (TLCA fed groups) were significantly higher (*P* < 0.05 in all cases); Chao1 index was significantly higher in TCDCA fed and TLCA fed groups (*P* < 0.05 in both cases), PD_whole_tree index was lower in the Chol group (*P* = 0.023). No significant difference in any of the diversity parameters was detected for the TUDCA fed group (**Figure 2A**).

For beta diversity, cluster analysis indicated that all samples were divided into two clades (**Figure 2D**), where the majority of samples from TCA fed, TCDCA fed, and TLCA fed groups clustered together. PCoA and PERMANOVA analyses with weighted_unifrac distance showed (**Supplementary Figure S2**) that TCA fed group was separated from the remaining groups (*F* > 7.11, *P* < 0.05 in all cases, **Supplementary Table S3**); TCDCA fed group was mixed with the TLCA fed group (*F* = 1.20, *P* = 0.2803), and separated from the remaining groups: TCA fed group (*F* = 7.11, *P* = 0.0118), TUDCA fed group (*F* = 9.79, *P* = 0.0058), Chol (*F* = 16.50, *P* = 0.0009), and Ctrl (*F* = 5.90, *P* = 0.0314). TUDCA fed group, TDCA fed group, Chol, and Ctrl groups could not be set apart from each other. In comparison to the Ctrl group, two primary BS groups (TCA fed and TCDCA fed groups) were significantly different (*P* = 0.0037 and *P* = 0.0314, respectively), whereas TUDCA fed group, Chol, and two secondary BA groups (TLCA fed and TDCA fed group) were not significantly different. Correlation analysis showed that *Cetobacterium* (*r* = -0.97, *P =* 0.000), *Bacteroides* (*r* = 0.75, *P =* 0.000), and Lachnospiraceae (*r* = 0.69, *P =* 0.000) contributed largely to the PCoA1 coordinate, whereas *Delftia* (*r* = -0.73, *P =* 0.000), Enterobacteriaceae (*r* = -0.70, *P =* 0.000), and *Bacteroides* (*r* = 0.63, *P =* 0.000) contributed largely to the PCoA2 coordinate (**Supplementary Table S4**).

In summary, primary BS (TCAS and TCDCAS) significantly decreased proportion of the Fusobacteria while promoted proportion of the Proteobacteria, and they also increased alpha diversity and induced community succession of the gut microbiota; Secondary BS (TLCAS and TDCAS) resulted in a higher F/B ratio, TLCAS also increased proportion of the Proteobacteria and Firmicutes. TUDCAS had no significant effect either on structure or F/B ratio of the gut microbiota.

### Differences in Taxonomic Abundance Among Groups

**FIGURE 3:**
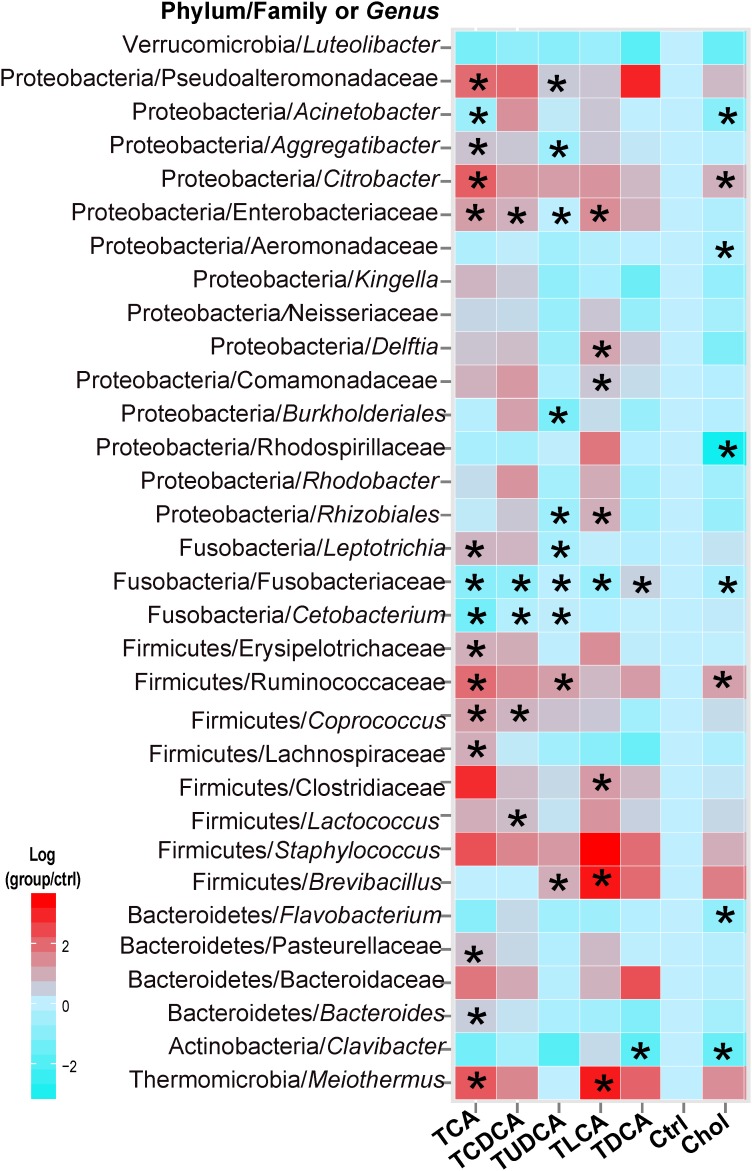
Thirty-two microbial taxa at the genus taxonomic level showed significant (*p* < 0.05) differences (in comparison to the Ctrl group) using the nonparametric Kruskal–Wallis test correcting for multiple comparisons with the Benjamini–Hochberg false discovery rate (FDR). Pairwise comparison of each group with the Ctrl group was tested using *t*-tests, and *P*-values < 0.05 are marked with “^∗^”. Where a taxon could not be precisely identified to the genus level, only a family name is indicated.

In the Kruskal–Wallis test of taxonomic abundance at the genus level, 32 bacterial genera exhibited significant differences among different groups (*P* < 0.05 in all cases, **Supplementary Table S5**; **Figure 3**). In comparison to the Ctrl group, and with the exception of TDCA fed group, the remaining groups exhibited varying degrees of decrease in Fusobacteriaceae (some genera could not be precisely identified in the analysis, so only the family name was assigned) and *Cetobacterium*. More specifically, 15 taxa, including Clostridiaceae, Ruminococcaceae, and *Citrobacter*, were significantly changed in the TCA fed group; 5 genera, including *Lactococcus* and *Coprococcus*, were significantly changed in the TCDCA fed group; 10 taxa, including Ruminococcaceae and *Citrobacter*, were significantly changed in the TUDCA fed group; 8 taxa, including *Brevibacillus*, Clostridiaceae, and *Meiothermus*, were significantly changed in the TLCA fed group; 2 taxons, including *Clavibacter* and Fusobacteriaceae, were significantly changed in the TDCA fed group; and 8 taxa, including Rhodospirillaceae and *Acinetobacter*, were significantly changed in the Chol group (*P* < 0.05 in all cases, **Supplementary Table S5**).

### Changes in KEGG Pathways Associated With Lipid Metabolism

**FIGURE 4:**
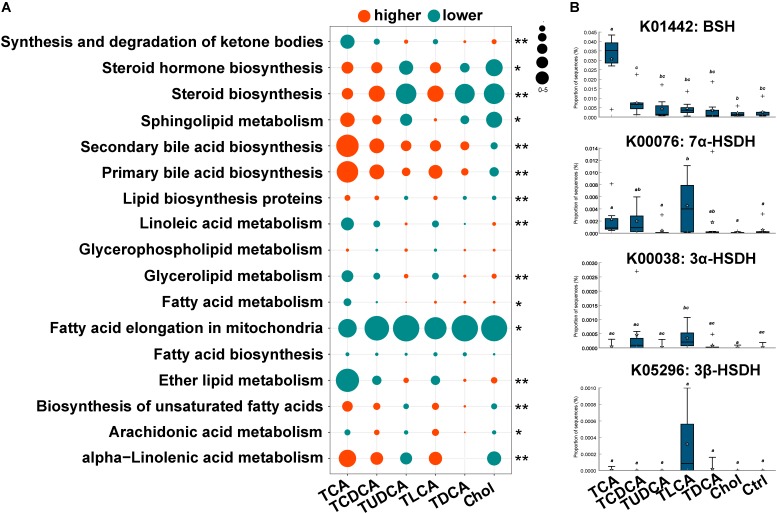
Changes in KEGG pathway and candidate genes between gut microbiota in the six experimental groups and the control group, predicted by PICRUST. **(A)** Changes in KEGG pathways associated with lipid metabolism. For each pathway in each group, log2 value of the proportion of functional shift is shown (represented by circle size and color). Net positive shifts (red) indicate increased pathway activity (higher) in a group. Net negative shifts (green) indicate lower pathway activity (lower) in a group. The size of circle corresponds to the magnitude of the shift. *P*-value was calculated using the nonparametric Kruskal–Wallis test correcting for multiple comparisons with the Benjamini–Hochberg false discovery rate (FDR); 0.001 < *P* < 0.05 values are marked with “^∗^” and *P* = 0.001 values with “^∗∗^”. **(B)** The relative abundance of four candidate genes involving in BA biotransformation of gut microbiota. Different letters above bars indicate statistically significant (*P* < 0.05) differences between groups. Groups not sharing the same lowercase letter are significantly different (*P* < 0.05).

The PICRUST prediction revealed that most KEGG pathways associated with microbial lipid metabolism were altered in the fish fed BS-supplemented diets (**Figure 4A**). Specifically, our results revealed a marked higher abundance in genes involved in the biosynthesis of secondary BAs (*P* = 0.000). Another notable observation was the significantly lower abundance of genes involved in fatty acid elongation pathway in mitochondria in all treatment groups (*P* = 0.000). Furthermore, genes in pathways associated with the biosynthesis of steroids (*P* = 0.011) and unsaturated fatty acids (*P* = 0.000), as well as metabolism of sphingolipids (*P* = 0.001) and alpha-linolenic acid (*P* = 0.000), were more abundant in TCA fed, TCDCA fed, and TLCA fed groups, and less abundant in the remaining three groups. Contrarily, genes in linoleic acid metabolism (*P* = 0.000) and glycerolipid metabolism (*P* = 0.000) were less abundant in the TCA fed, TCDCA fed, and TLCA fed groups, and more abundant in the remaining three groups. As regards, the microbial genes involved in biomodification of BAs, the abundance of BSH was highly significantly increased in the TCA fed group (*P* = 0.01), and the abundance of 7α-HSDH (*P* = 0.020), 3α-HSDH (*P* = 0.053), and 3β-HSDH (*P* = 0.056) increased in the TLCA fed group (all compared to the Control group; **Figure 4B**).

### Association Between Biliary BAs and Gut Microbiota

**FIGURE 5:**
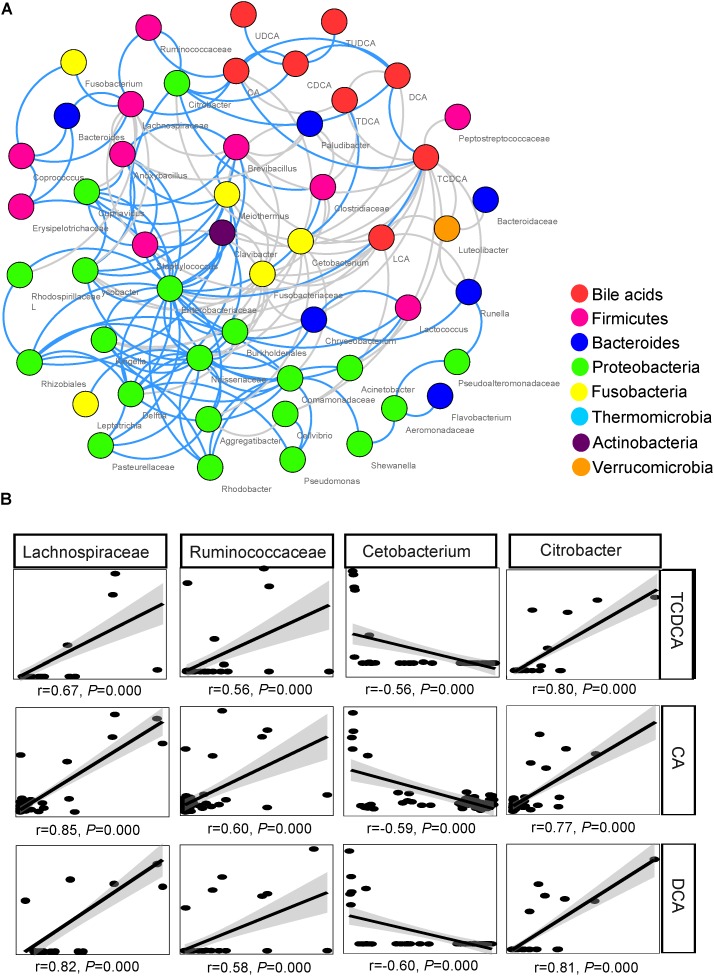
Association between biliary BAs and taxa of gut microbiota. **(A)** Molecular ecological network (MEN) of biliary BAs and the top 50 microbial taxa at the genus level. A gray line indicates a negative correlation between two individual nodes, and a blue line a positive correlation. **(B)** Pearson correlations between three biliary acids and four key taxa of gut microbiota.

The network inferred through MENA analysis showed that eight biliary BAs and 43 microbial taxa were involved in the interplay between biliary BAs and gut microbiota. These BAs were CA, CDCA, TCDCA, DCA, TDCA, LCA, UDCA, and TUDCA. Microbial taxa included 10 taxa from Firmicutes, 6 taxa from Bacteroides, 20 taxa from Proteobacteria, 4 taxa from Fusobacteria, 1 taxon from Thermomicrobia, 1 taxon from Actinobacteria, and 1 taxon from Verrucomicrobia. In the Firmicutes, Lachnospiraceae, Ruminococcaceae, Clostridiaceae, and *Lactococcus* were directly correlated with BAs; no Bacteroides taxon was directly correlated with BAs; only 2 among the 21 Proteobacteria taxa were directly correlated with BAs: *Citrobacter* and *Acinetobacter* (**Figure 5A**).

Among the microbial taxa directly correlated with BAs, Lachnospiraceae, Ruminococcaceae, and *Citrobacter*, were strongly correlated with the concentration of biliary CA, with *r* = 0.85, 0.60, and 0.77, respectively (*P* < 0.001 in all cases, **Figure 5B**; **Table 2**). These three taxa also exhibited strong correlations with the concentrations of DCA and TCDCA. *Bacteroides* and *Coprococcus* genera exhibited moderate correlations with the concentrations of CA (*r* = 0.55, *P* = 0.001 and *r* = 0.50, *P* = 0.001, respectively), DCA (*r* = 0.51, *P* = 0.001 and *r* = 0.47, *P* = 0.001, respectively), and TCDCA (*r* = 0.31, *P* = 0.004 and *r* = 0.41, *P* = 0.003, respectively). The concentration of CDCA was correlated with the abundance of Lachnospiraceae (*r* = 0.42, *P* = 0.003), *Coprococcus* (*r* = 0.33, *P* = 0.027), Ruminococcaceae (*r* = 0.44, *P* = 0.001), and *Citrobacter* (*r* = 0.50, *P* = 0.000); LCA with Lachnospiraceae (*r* = 0.32, *P* = 0.030); TCA with *Anoxybacillus* (*r* = 0.58, *P* = 0.000), *Lysobacter* (*r* = 0.58, *P* = 0.000), *Cupriavidus* (*r* = 0.56, *P* = 0.001), Rhodospirillaceae (*r* = 0.55, *P* = 0.001), *Brevibacillus* (*r* = 0.50, *P* = 0.010), and *Lactococcus* (*r* = 0.47, *P* = 0.010). Beside these interactions, we also found some weak correlations: Clostridiaceae was weakly related to the concentration of LCA (*r* = 0.31, *P =* 0.028), CA (*r* = 0.36, *P =* 0.011), and DCA (*r* = 0.33, *P =* 0.038). A negative relationship between *Cetobacterium* and the concentration of biliary CA (*r* = -0.59, *P =* 0.000), DCA (*r* = -0.60, *P =* 0.000), and TCDCA (*r* = -0.56, *P =* 0.000) was also detected.

**Table 2:** Pearson correlations between biliary acids and gut microbiota taxa at the genus level (*r* value).

	TCA	TCDCA	TUDCA	TDCA	TLCA	CA	CDCA	UDCA	DCA	LCA
*Bacteroides*	–0.01	**0.31**	0.22	–0.23	0.21	**0.55**	**0.28**	0.07	**0.51**	0.26
*Anoxybacillus*	**0.58**	–0.07	–0.14	0.03	–0.02	–0.11	–0.20	–0.21	–0.08	–0.05
*Brevibacillus*	**0.50**	–0.07	–0.13	0.11	–0.05	–0.10	–0.15	–0.19	–0.08	–0.04
*Lactococcus*	**0.47**	0.12	–0.05	–0.01	0.20	0.03	–0.15	–0.15	0.04	0.08
Clostridiaceae	–0.03	**0.54**	–0.02	–0.10	**0.40**	**0.36**	0.06	0.14	**0.33**	0.31
Lachnospiraceae	–0.01	**0.67**	0.19	–0.23	0.29	**0.85**	**0.42**	0.23	**0.82**	**0.32**
*Coprococcus*	0.10	**0.41**	0.15	–0.22	0.17	**0.50**	**0.33**	0.10	**0.47**	0.01
Ruminococcaceae	–0.02	**0.56**	**0.39**	–0.10	0.15	**0.60**	**0.44**	**0.33**	**0.58**	–0.01
*Cetobacterium*	–0.13	–**0.56**	–0.06	0.24	–**0.37**	–**0.59**	–0.17	0.02	–**0.60**	–0.24
Rhodospirillaceae	**0.55**	–0.06	–0.10	–0.04	0.01	–0.08	–0.17	–0.17	–0.07	–0.02
*Cupriavidus*	**0.56**	–0.07	–0.15	0.07	0.03	–0.11	–0.19	–0.22	–0.08	–0.01
*Citrobacter*	0.08	**0.80**	0.16	–0.18	0.20	**0.77**	**0.50**	0.30	**0.81**	0.06
*Lysobacter*	**0.58**	–0.05	–0.13	0.07	–0.03	–0.10	–0.18	–0.21	–0.07	–0.04


*P-values < 0.05 are bolded. Where a taxon could not be precisely identified to the genus level, only a family name is indicated.*

## Discussion

Although the interaction between BAs and gut microbiota has received ample scientific attention (Yokota et al., 2012; Sayin et al., 2013; Ridlon et al., 2014), *in vivo* experiments focusing on both the fluctuation of biliary BAs and gut microbiota responses to different BS supplemented in diet remain exceedingly rare. Although impacts of different BS dietary supplementations on regulation of BA synthesis have been studied in rats (Heuman et al., 1988; Ren et al., 2003), these studies ignored their influence on gut microbiota. Two *in vivo* studies on mice have shown that the regulation of BAs affects gut microbiota, but both studied only the effects of CA (Islam et al., 2011; Zheng et al., 2017). Therefore, it appears that ours could be the first attempt to study this topic in non-mammalian animals, as well as the first attempt to precisely elucidate the interactions between different dietary BAs, biliary Bas, and gut microbiota in fish.

Our results revealed a notable similarity in the effects of dietary BS on shaping the host’s BA pool between grass carp and mammals with several evidences. We found that TCAS resulted in extremely high levels of CA and DCA, which resembles the effects of supplementing CA in rat diet (Islam et al., 2011). Supplementation of TUDCAS and TCDCAS resulted in a moderate increase in biliary CA, which indicates that both TCDCAS and TUDCAS can regulate the biosynthesis of primary BAs in fish. In human clinical treatments, UDCA and CDCA are used as enterohepatic drugs that can regulate BA biosynthesis and increase the level of primary BAs (Hofmann, 1994; Sinakos et al., 2010). Cholestyramine resin can also upregulate the synthesis of BAs in humans (Einarsson et al., 1991). We also observed a slight increase of CA and CDCA in the cholestyramine resin group (Chol).

Our result also confirmed that CA, as an important factor regulating the gut microbiota in rats (Islam et al., 2011; Zheng et al., 2017), has similar impacts in grass carp. Apart from this, we provided a more detailed information about functions of different BS in the regulation of gut microbiota. We found that two primary BS (TCAS and TCDCAS) tended to increase alpha diversity and induce population successions of gut microbiota, whereas the secondary BS (TDCAS and TLCAS) resulted in an increase of the F/B ratio. Intriguingly, TUDCAS did not exhibit any of these effects. The change of gut microbiota would resulted in functional alteration. Regarding BAs biotransformation effect of the gut microbiota, our results showed that BSHs of the gut microbiota, which are key enzymes in BS deconjugation (Ridlon et al., 2006), were more abundant in the TCA fed group (**Figure 4B**). This result was consistent evidence that the level of free CA with was higher than conjugated TCA in TCA fed group and corroborated that the gut microbiota in grass carp intestine took part in deconjugation of the BAs. The same evidence was also found in TCDCA fed group where the level of free CDCA was higher than conjugated TCDCA and a higher level of the BSHs in the gut microbiota accompanied. Otherwise, the higher abundance of 7α-HSDHs and 7α-HSDH in TLCA fed group suggested an altered effect of its gut microbiota on BA oxidation and epimerization (Ridlon et al., 2006). Another interesting evidence was that level of the biliary TUDCA was promoted in TCDCA fed group. The conservation of CDCA to UDCA has been observed in human gut microbiota (Fedorowski et al., 1979). In our experiment, it could be possible that TCDCA was converted to TUDCA, but we need more evidences to prove this point. Anyway, it is still unclear whether UDCA is a primary or a senconday BA in grass carp.

Our experiments showed that dietary BS affect composition of the gut microbiota, their capacity for BA biotransformation (with changed proportion of BSHs and HSDHs as the evidence) and biosynthesis of secondary BAs. In turn, this change in the rate of BA biotransformation might regulate the biosynthesis of primary BAs in liver (Ridlon et al., 2014). We found some indications for the alteration of primary BA biosythesis rate: the increased level of TUDCA and CDCA in TCA fed group, and the increased level of CA in TUDCA fed and TCDCA fed groups. Except for these effects on BA biosynthesis, dietary BS-triggered composition variation of gut microbiota was also reflected in other aspects of lipid metabolism. Although different dietary BS produced the same effect on fatty acid elongation in mitochondria, two groups of BS (TCAS, TCDCAS, and TLCAS vs. TDCAS and TUDCAS) produced opposite effects on the metabolism of steroids, unsaturated fatty acids, sphingolipids, alpha-linolenic acid, linoleic acid, and glycerolipids.

Age (Mariat et al., 2009), diet (Turnbaugh et al., 2006; Albenberg and Wu, 2014), and metabolic syndromes, such as obesity (Labus et al., 2017), are known to be important modulatory factors of the F/B ratio. The so called “obese microbiota,” which appear to extract more energy from a diet (Turnbaugh et al., 2006), are usually associated with higher F/B ratios. Herein, we found that the addition of two secondary BS (TLCAS and TDCAS) has resulted in significantly higher F/B ratios, which indicates that dietary secondary BS were also modulatory factors of the F/B ratio. Otherwise, we also found that all groups with high F/B ratios (TCA fed, TLCA fed, and TDCA fed groups) had significantly higher levels of secondary BAs in the gall. It is known that secondary BAs are barely reabsorbed in the intestinal epithelium and their concentrations are extremely low in the gall under normal conditions (Hofmann, 2009; Hofmann and Hagey, 2014). Therefore, the higher level of secondary BAs in the gall indicates a change in their reabsorption rate in the intestinal epithelium. Gut microbiota could influence intestinal epithelial permeability and inflammation and higher F/B ratio was usually associated with higher epithelial permeability in guts (Cani et al., 2008). These observations suggested that gut microbiota (F/B ratio) or their metabolites (secondary BS) probably changed the permeability of the intestinal epithelium and the reabsorption of secondary BAs in the gut.

TCAS and TCDCAS significantly decreased the proportion of Fusobacteria, which is the dominant bacterial taxon among the fish gut microbiota (Larsen et al., 2014; Yan et al., 2016), in our experiment (**Supplementary Figure S1**). TCAS, TCDCAS, and TLCAS significantly increased the proportion of Proteobacteria as well as its member taxon, Pseudoalteromonadaceae (**Supplementary Figure S1** and **Figure 3**). The interaction network among biliary BAs and gut microbiota showed that CA and TCDCA were positively related to the proportion of most Proteobacteria taxa (**Figure 5A**). Fish bacterial diseases are usually caused by the Proteobacteria genera, such as *Pseudomonas* (Wu et al., 2012), and Proteobacteria can also cause inflammation in humans. In this aspect, BAs, especially CA and CDCA, might increase the probability of bacterial infection in fish. *Bacteroides*, Lachnospiraceae, Ruminococcaceae, and *Citrobacter* were the bacterial taxa most closely positively correlated with biliary BAs in our experiment. Ruminococcaceae are known to be involved in the biotransformation of primary BAs into secondary BAs in mammals (Ridlon et al., 2006). This taxon was also positively correlated with CA and DCA in rats (Islam et al., 2011), which is consistent with our results. However, *Bacteroides* was negatively correlated with BAs in rats, which might be due to different hosts and diets. The interaction between Lachnospiraceae and BAs, however, is a novel finding. This might also be species-specific, as a central role of this taxon in the regulation of microbial populations in the guts of grass carp has been proposed before (Hao et al., 2017). These four bacterial taxa, closely related with biliary BAs in our study, have been associated with obesity and a number of metabolic syndromes; for example: an increased ratio of Lachnospiraceae has influenced the development of obesity and diabetes in mice with genetic susceptibility for obesity (Kameyama and Itoh, 2014), Lachnospiraceae and *Bacteroides* are associated with diabetes in humans (Brugman et al., 2006), the Ruminococcaceae family is negatively related to cirrhosis and insulin secretion in humans (Bajaj et al., 2014; O’Connor et al., 2014), and its abundance usually increases in response to high-fat diets (Kim et al., 2012; Daniel et al., 2014). Therefore, there are indications that changes of gut microbiota triggered by dietary BSs observed in this study might result in increased synthesis of toxic secondary BAs and increased incidence of obesity.

Overall, our results revealed a notable similarity in the effects of dietary BS on shaping the host’s BA pool between grass carp and mammals. This indicates a high level of conservation in BA metabolism between these two evolutionary (relatively) distant groups of animals. Our results also revealed the distinct effects of different dietary BAs on gut microbiota. TUDCAS had no significant effect on composition of the gut microbiota in grass carp. The changes of the gut microbiota resulted in alteration in BAs biotransformation and increased synthesis of toxic secondary BAs. These results suggested that UDCA should be more appropriate for treatment of grass carp hepatobiliary disease because it had lesser side effects.

## Author Contributions

S-GW and G-TW were the main contributors of the manuscript. S-GW and G-TW contributed to the study design, the acquisition of the funding, the oversight of the study, the interpretation of the data, and the editing of the manuscript. FX was responsible for the design, the data collection, the analysis, the interpretation of data, and the writing of the manuscript. S-GW and JZ contributed to the collection, the analysis, and the interpretation of the data. IJ was responsible for the quality control, the interpretation of the data, and language editing of the manuscript. HZ was responsible for the study coordination and quality control, and the editing of the manuscript. W-XL and ML contributed to the fieldwork data collection and the drafting of the manuscript.

## Conflict of Interest Statement

The authors declare that the research was conducted in the absence of any commercial or financial relationships that could be construed as a potential conflict of interest.

## Footnotes

1 http://qiime.org/

## References

[B1] Albenberg L. G. Wu G. D. (2014). Diet and the intestinal microbiome: associations, functions, and implications for health and disease. *Gastroenterology* 146 1564–1572. 10.1053/j.gastro.2014.01.058 24503132 PMC4216184

[B2] Bajaj J. S. Heuman D. M. Hylemon P. B. Sanyal A. J. White M. B. Monteith P. (2014). Altered profile of human gut microbiome is associated with cirrhosis and its complications. *J. Hepatol.* 60 940–947. 10.1016/j.jhep.2013.12.019 24374295 PMC3995845

[B3] Berr F. Kullak-Ublick G. A. Paumgartner G. Munzing W. Hylemon P. B. (1996). 7 alpha-dehydroxylating bacteria enhance deoxycholic acid input and cholesterol saturation of bile in patients with gallstones. *Gastroenterology* 111 1611–1620. 10.1016/S0016-5085(96)70024-0 8942741

[B4] Brugman S. Klatter F. Visser J. Wildeboer-Veloo A. Harmsen H. Rozing J. (2006). Antibiotic treatment partially protects against type 1 diabetes in the bio-breeding diabetes-prone rat. Is the gut flora involved in the development of type 1 diabetes? *Diabetologia* 49 2105–2108. 10.1007/s00125-006-0334-0 16816951

[B5] Cani P. D. Bibiloni R. Knauf C. Waget A. Neyrinck A. M. Delzenne N. M. (2008). Changes in gut microbiota control metabolic endotoxemia-induced inflammation in high-fat diet-induced obesity and diabetes in mice. *Diabetes Metab. Res. Rev.* 57 1470–1481. 10.2337/db07-1403 18305141

[B6] Caporaso J. G. Kuczynski J. Stombaugh J. Bittinger K. Bushman F. D. Costello E. K. (2010). QIIME allows analysis of high-throughput community sequencing data. *Nat. Methods* 7 335–336. 10.1038/nmeth.f.303 20383131 PMC3156573

[B7] Caporaso J. G. Lauber C. L. Walters W. A. Berg-Lyons D. Lozupone C. A. Turnbaugh P. J. (2011). Global patterns of 16S rRNA diversity at a depth of millions of sequences per sample. *Proc. Natl. Acad. Sci. U.S.A.* 108 4516–4522. 10.1073/pnas.1000080107 20534432 PMC3063599

[B8] Chen H. J. (2016). *Influence of Bile Acids on the Growth Performance, Lipid Metabolism and the Intestinal Microbiota in Grass Carp, Ctenopharyngodon Idella.* Master’s thesis, North West Agriculture and Forestry University, Shaanxi.

[B9] Claesson M. J. Jeffery I. B. Conde S. Power S. E. O′connor E. M. Cusack S. (2012). Gut microbiota composition correlates with diet and health in the elderly. *Nature* 492 178–184. 10.1038/nature11319 22797518

[B10] D’Aldebert E. Mve M. J. B. B. Mergey M. Wendum D. Coilly A. Fouassier L. (2009). Bile salts control the antimicrobial peptide cathelicidin through nuclear receptors in the human biliary epithelium. *Gastroenterology* 136 1435–1443. 10.1053/j.gastro.2008.12.040 19245866

[B11] Daniel H. Gholami A. M. Berry D. Desmarchelier C. Hahne H. Loh G. (2014). High-fat diet alters gut microbiota physiology in mice. *ISME J.* 8 295–308. 10.1038/ismej.2013.155 24030595 PMC3906816

[B12] Deng Y. Jiang Y. H. Yang Y. He Z. Luo F. Zhou J. (2012). Molecular ecological network analyses. *BMC Bioinformatics* 13:113. 10.1186/1471-2105-13-113 22646978 PMC3428680

[B13] DeSantis T. Z. Hugenholtz P. Larsen N. Rojas M. Brodie E. L. Keller K. (2006). Greengenes, a chimerachecked 16S rRNA gene database and workbench compatible with ARB. *Appl. Environ. Microb.* 72 5069–5072. 10.1128/AEM.03006-05 16820507 PMC1489311

[B14] Edgar R. C. (2010). Search and clustering orders of magnitude faster than BLAST. *Bioinformatics* 26 2460–2461. 10.1093/bioinformatics/btq461 20709691

[B15] Edgar R. C. Haas B. J. Clemente J. C. Quince C. Knight R. (2011). UCHIME improves sensitivity and speed of chimera detection. *Bioinformatics* 27 2194–2200. 10.1093/bioinformatics/btr381 21700674 PMC3150044

[B16] Einarsson K. Ericsson S. Ewerth S. Reihner E. Rudling M. Ståhlberg D. (1991). Bile acid sequestrants: mechanisms of action on bile acid and cholesterol metabolism. *Eur. J. Clin. Pharmacol.* 40 53–58. 10.1007/BF01409410 2044645

[B17] Faoro H. (2015). *FAO Yearbook: Fisheries and Aquaculture Statistics.* Rome: Food and Agriculture Organization of the United Nations.

[B18] Fedorowski T. Salen G. Tint G. S. Mosbach E. (1979). Transformation of chenodeoxycholic acid and ursodeoxycholic acid by human intestinal bacteria. *Gastroenterology* 77 1068–1073. 488633

[B19] Gilbert J. A. Field D. Swift P. Newbold L. Oliver A. Smyth T. (2009). The seasonal structure of microbial communities in the Western English Channel. *Environ. Microbiol.* 11 3132–3139. 10.1111/j.1462-2920.2009.02017.x 19659500

[B20] Hagio M. Matsumoto M. Ishizuka S. (2011). “Bile acid analysis in various biological samples using ultra performance liquid chromatography/electrospray ionization-mass spectrometry (UPLC/ESI-MS),” in *Metabolic Profiling: Methods and Protocols*, ed. Metz T. O. (Totowa, NJ: Humana Press), 119–129. 10.1007/978-1-61737-985-7_6 21207286

[B21] Hao Y. T. Wu S. G. Xiong F. Tran N. T. Jakovlić I. Zou H. (2017). Succession and fermentation products of grass carp (*Ctenopharyngodon idellus*) hindgut microbiota in response to an extreme dietary shift. *Front. Microbiol.* 8:1585. 10.3389/fmicb.2017.01585 28871246 PMC5566599

[B22] Heuman D. M. Vlahcevic Z. Bailey M. L. Hylemon P. B. (1988). Regulation of bile acid synthesis. II. Effect of bile acid feeding on enzymes regulating hepatic cholesterol and bile acid synthesis in the rat. *Hepatology* 8 892–897. 10.1002/hep.1840080431 3391517

[B23] Hofmann A. (1994). Pharmacology of ursodeoxycholic acid, an enterohepatic drug. *Scand. J. Gastroenterol. Suppl.* 29 1–15. 10.3109/00365529409103618 7824870

[B24] Hofmann A. F. (1999). The continuing importance of bile acids in liver and intestinal disease. *Arch. Intern. Med.* 159 2647–2658. 10.1001/archinte.159.22.2647 10597755

[B25] Hofmann A. F. (2009). The enterohepatic circulation of bile acids in mammals: form and functions. *Front. Biosci.* 14:2584–2598. 10.2741/3399 19273221

[B26] Hofmann A. F. Hagey L. R. (2014). Bile acid chemistry, biology, and therapeutics during the last 80 years: historical aspects. *J. Lipid Res.* 55 1553–1595. 24838141 10.1194/jlr.R049437 PMC4109754

[B27] Inagaki T. Moschetta A. Lee Y. K. Peng L. Zhao G. Downes M. (2006). Regulation of antibacterial defense in the small intestine by the nuclear bile acid receptor. *Proc. Natl. Acad. Sci. U.S.A.* 103 3920–3925. 10.1073/pnas.0509592103 16473946 PMC1450165

[B28] Islam K. B. Fukiya S. Hagio M. Fujii N. Ishizuka S. Ooka T. (2011). Bile acid is a host factor that regulates the composition of the cecal microbiota in rats. *Gastroenterology* 141 1773–1781. 10.1053/j.gastro.2011.07.046 21839040

[B29] Kameyama K. Itoh K. (2014). Intestinal colonization by a Lachnospiraceae bacterium contributes to the development of diabetes in obese mice. *Microbes Environ.* 29 427–430. 10.1264/jsme2.ME14054 25283478 PMC4262368

[B30] Kelly B. J. Gross R. Bittinger K. Sherrill-Mix S. Lewis J. D. Collman R. G. (2015). Power and sample-size estimation for microbiome studies using pairwise distances and PERMANOVA. *Bioinformatics* 31 2461–2468. 10.1093/bioinformatics/btv183 25819674 PMC4514928

[B31] Kim K. A. Gu W. Lee I. A. Joh E. H. Kim D. H. (2012). High fat diet-induced gut microbiota exacerbates inflammation and obesity in mice via the TLR4 signaling pathway. *PLoS One* 7:e47713. 10.1371/journal.pone.0047713 23091640 PMC3473013

[B32] Kurdi P. Kawanishi K. Mizutani K. Yokota A. (2006). Mechanism of growth inhibition by free bile acids in lactobacilli and bifidobacteria. *J. Bacteriol.* 188 1979–1986. 10.1128/JB.188.5.1979-1986.2006 16484210 PMC1426545

[B33] Kurumiya Y. Nagino M. Nozawa K. Kamiya J. Uesaka K. Sano T. (2003). Biliary bile acid concentration is a simple and reliable indicator for liver function after hepatobiliary resection for biliary cancer. *Surgery* 133 512–520. 10.1067/msy.2003.142 12773979

[B34] Labus J. S. Hollister E. B. Jacobs J. Kirbach K. Oezguen N. Gupta A. (2017). Differences in gut microbial composition correlate with regional brain volumes in irritable bowel syndrome. *Microbiome* 5:49. 10.1186/s40168-017-0260-z 28457228 PMC5410709

[B35] Langille M. G. I. Zaneveld J. Caporaso J. G. McDonald D. Knights D. Reyes J. A. (2013). Predictive functional profiling of microbial communities using 16S rRNA marker gene sequences. *Nat. Biotechnol.* 31 814–821. 10.1038/nbt.2676 23975157 PMC3819121

[B36] Larsen A. M. Mohammed H. H. Arias C. R. (2014). Characterization of the gut microbiota of three commercially valuable warm water fish species. *J. Appl. Microbiol.* 116 1396–1404. 10.1111/jam.12475 24529218

[B37] Lee C. H. Olson P. Evans R. M. (2003). Minireview: lipid metabolism, metabolic diseases, and peroxisome proliferator-activated receptors. *Endocrinology* 144 2201–2207. 10.1210/en.2003-0288 12746275

[B38] Ley R. E. Turnbaugh P. J. Klein S. Gordon J. I. (2006). Microbial ecology: human gut microbes associated with obesity. *Nature* 444 1022–1023. 10.1038/4441022a 17183309

[B39] Li W. Godzik A. (2006). Cd-hit: a fast program for clustering and comparing large sets of protein or nucleotide sequences. *Bioinformatics* 22 1658–1659. 10.1093/bioinformatics/btl158 16731699

[B40] Liu H. Hu C. Zhang X. Jia W. (2017). Role of gut microbiota, bile acids and their cross-talk in the effects of bariatric surgery on obesity and type 2 diabetes. *J. Diabetes Investig.* 9 13–20. 10.1111/jdi.12687 28434196 PMC5754516

[B41] Magoc T. Salzberg S. L. (2011). FLASH: fast length adjustment of short reads to improve genome assemblies. *Bioinformatics* 27 2957–2963. 10.1093/bioinformatics/btr507 21903629 PMC3198573

[B42] Mariat D. Firmesse O. Levenez F. Guimaraes V. Sokol H. Dore J. (2009). The Firmicutes/Bacteroidetes ratio of the human microbiota changes with age. *BMC Microbiol.* 9:123. 10.1186/1471-2180-9-123 19508720 PMC2702274

[B43] Navas-Molina J. A. Peralta-Sanchez J. M. Gonzalez A. McMurdie P. J. Vazquez-Baeza Y. Xu Z. J. (2013). Advancing our understanding of the human microbiome using QIIME. *Method. Enzymol.* 531 371–444. 10.1016/B978-0-12-407863-5.00019-8 24060131 PMC4517945

[B44] Ni D. S. Wang J. G. (1999). *Biology and Diseases of Grass Carp.* Beijing: Science Press.

[B45] Nittono H. (1980). Bile acid metabolism in infants and children and its implications for hepatobiliary diseases. *Indian J. Pediatr.* 47 549–553. 10.1007/BF02822549 7262972

[B46] O’Connor A. Quizon P. M. Albright J. E. Lin F. T. Bennett B. J. (2014). Responsiveness of cardiometabolic-related microbiota to diet is influenced by host genetics. *Mamm. Genome* 25 583–599. 10.1007/s00335-014-9540-0 25159725 PMC4239785

[B47] Parks D. H. Tyson G. W. Hugenholtz P. Beiko R. G. (2014). STAMP: statistical analysis of taxonomic and functional profiles. *Bioinformatics* 30 3123–3124. 10.1093/bioinformatics/btu494 25061070 PMC4609014

[B48] R Core Team (2014). *R: A Language and Environment for Statistical Computing.* Vienna: R Foundation for Statistical Computing.

[B49] Reddy J. K. Rao M. S. (2006). Lipid metabolism and liver inflammation. II. Fatty liver disease and fatty acid oxidation. *Am. J. Physiol. Gastrointest. Liver Physiol.* 290 852–858. 10.1152/ajpgi.00521.2005 16603729

[B50] Ren S. Marques D. Redford K. Hylemon P. B. Gil G. Vlahcevic Z. R. (2003). Regulation of oxysterol 7α-hydroxylase (CYP7B1) in the rat. *Metabolism* 52 636–642. 10.1053/meta.2003.50106 12759897

[B51] Ridlon J. M. Kang D. J. Hylemon P. B. (2006). Bile salt biotransformations by human intestinal bacteria. *J. Lipid Res.* 47 241–259. 10.1194/jlr.R500013-JLR200 16299351

[B52] Ridlon J. M. Kang D. J. Hylemon P. B. Bajaj J. S. (2014). Bile acids and the gut microbiome. *Curr. Opin. Gastroenterol.* 30 332–338. 10.1097/MOG.0000000000000057 24625896 PMC4215539

[B53] Russell D. W. (2003). The enzymes, regulation, and genetics of bile acid synthesis. *Annu. Rev. Biochem.* 72 137–174. 10.1146/annurev.biochem.72.121801.161712 12543708

[B54] Sayin S. I. Wahlström A. Felin J. Jäntti S. Marschall H. U. Bamberg K. (2013). Gut microbiota regulates bile acid metabolism by reducing the levels of tauro-beta-muricholic acid, a naturally occurring FXR antagonist. *Cell Metab.* 17 225–235. 10.1016/j.cmet.2013.01.003 23395169

[B55] Shannon P. Markiel A. Ozier O. Baliga N. S. Wang J. T. Ramage D. (2003). Cytoscape: a software environment for integrated models of biomolecular interaction networks. *Genome Res.* 13 2498–2504. 10.1101/gr.1239303 14597658 PMC403769

[B56] Sinakos E. Marschall H. U. Kowdley K. V. Befeler A. Keach J. Lindor K. (2010). Bile acid changes after high-dose ursodeoxycholic acid treatment in primary sclerosing cholangitis: relation to disease progression. *Hepatology* 52 197–203. 10.1002/hep.23631 20564380 PMC2928060

[B57] Swann J. R. Want E. J. Geier F. M. Spagou K. Wilson I. D. Sidaway J. E. (2011). Systemic gut microbial modulation of bile acid metabolism in host tissue compartments. *Proc. Natl. Acad. Sci. U.S.A.* 108 4523–4530. 10.1073/pnas.1006734107 20837534 PMC3063584

[B58] Tremaroli V. Bäckhed F. (2012). Functional interactions between the gut microbiota and host metabolism. *Nature* 489 242–249. 10.1038/nature11552 22972297

[B59] Turnbaugh P. J. Ley R. E. Mahowald M. A. Magrini V. Mardis E. R. Gordon J. I. (2006). An obesity-associated gut microbiome with increased capacity for energy harvest. *Nature* 444 1027–1031. 10.1038/nature05414 17183312

[B60] Vallim T. Q. D. A. Tarling E. J. Edwards P. A. (2013). Pleiotropic roles of bile acids in metabolism. *Cell Metab.* 17 657–669. 10.1016/j.cmet.2013.03.013 23602448 PMC3654004

[B61] Wu S. Wang G. Angert E. R. Wang W. Li W. Zou H. (2012). Composition, diversity, and origin of the bacterial community in grass carp intestine. *PLoS One* 7:e30440. 10.1371/journal.pone.0030440 22363439 PMC3282688

[B62] Yan Q. Li J. Yu Y. Wang J. He Z. Van Nostrand J. D. (2016). Environmental filtering decreases with fish development for the assembly of gut microbiota. *Environ. Microbiol.* 18 4739–4754. 10.1111/1462-2920.13365 27130138

[B63] Yokota A. Fukiya S. Islam K. B. Ooka T. Ogura Y. Hayashi T. (2012). Is bile acid a determinant of the gut microbiota on a high-fat diet? *Gut Microbes* 3 455–459. 10.4161/gmic.21216 22825495

[B64] Yoshimoto S. Loo T. M. Atarashi K. Kanda H. Sato S. Oyadomari S. (2013). Obesity-induced gut microbial metabolite promotes liver cancer through senescence secretome. *Nature* 499 97–98. 10.1038/nature12347 23803760

[B65] Zhang J. Xiong F. Wang G. T. Li W. X. Li M. Zou H. (2017). The influence of diet on the grass carp intestinal microbiota and bile acids. *Aquac. Res.* 48 4934–4944. 10.1111/are.13312

[B66] Zhang Z. Li D. Refaey M. M. Xu W. (2017). High spatial and temporal variations of microbial community along the southern catfish gastrointestinal tract: insights into dynamic food digestion. *Front. Microbiol.* 8:1531. 10.3389/fmicb.2017.01531 28848535 PMC5552716

[B67] Zheng X. Huang F. Zhao A. Lei S. Zhang Y. Xie G. (2017). Bile acid is a significant host factor shaping the gut microbiome of diet-induced obese mice. *BMC Biol.* 15:120. 10.1186/s12915-017-0462-7 29241453 PMC5731064

[B68] Zhou H. Hylemon P. B. (2014). Bile acids are nutrient signaling hormones. *Steroids* 86 62–68. 10.1016/j.steroids.2014.04.016 24819989 PMC4073476

