# Supplementary material for: Dietary Bile Salt Types Influence the Composition of Biliary Bile Acids and Gut Microbiota in Grass Carp
Source: Front Microbiol. 2018 Sep 18;9:2209. doi: 10.3389/fmicb.2018.02209 (PMC6154720; doi:10.3389/fmicb.2018.02209)
Supplement: Supplementary file 1 [file Data_Sheet_1.PDF]

Supplemental material

Table S1. *P* values with concentration of each biliary BA between groups.

	Total BAs	CA	TUDCA	CDCA	TCDCA	DCA	TCA	TDCA	UDCA	LCA	TLCA
TCAvsTCDCA	**0.0003**	**0.0002**	0.1394	0.1275	**0.0084**	**0.0006**	0.1718	**0.0122**	**0.0131**	0.1505	0.9217
TCAvsTUDCA	**0.0002**	**0.0002**	0.2807	0.1875	**0.0084**	**0.0006**	0.7499	**0.0345**	0.6825	0.4882	0.1747
TCAvsTLCA	**0.0001**	**0.0002**	**0.0119**	**0.0233**	**0.0086**	**0.0006**	0.2949	**0.0128**	**0.0043**	0.5096	0.7612
TCAvsTDCA	**0.0001**	**0.0002**	0.3706	0.3900	**0.0085**	**0.0006**	**0.0293**	**0.0201**	0.8350	0.0541	0.1747
TCAvsCtrl	**0.0001**	**0.0002**	**0.0110**	**0.0196**	**0.0084**	**0.0006**	**0.0242**	**0.0150**	**0.0056**	0.9477	0.1747
TCAvsChol	**0.0001**	**0.0003**	**0.0069**	**0.0184**	**0.0084**	**0.0006**	0.2540	0.1300	**0.0171**	0.0541	0.1747
TCDCAvsTUDCA	0.5032	0.2862	0.5993	0.6890	1.0000	0.3821	0.1302	0.1199	0.0602	0.4377	0.0907
TCDCAvsTLCA	**0.0087**	**0.0208**	**0.0101**	**0.0072**	0.0828	0.8585	0.7005	0.1271	0.1684	0.3536	0.8053
TCDCAvsTDCA	0.0567	0.0887	0.0571	0.3975	0.3506	0.5160	0.1878	0.4009	**0.0346**	**0.0460**	0.0907
TCDCAvsCtrl	**0.0075**	**0.0119**	**0.0101**	**0.0041**	1.0000	0.2823	0.1301	0.6618	0.1410	0.1522	0.0907
TCDCAvsChol	**0.0311**	0.3370	**0.0064**	0.0945	1.0000	0.1513	0.6527	0.5053	0.8104	**0.0460**	0.0907
TUDCAvsTLCA	**0.0041**	**0.0217**	**0.0107**	**0.0134**	0.0828	0.2927	0.2990	0.4851	**0.0177**	0.9465	0.1705
TUDCAvsTDCA	0.0908	0.2446	0.1067	0.5956	0.3506	0.8380	**0.0021**	0.2305	0.8481	0.1223	1.0000
TUDCAvsCtrl	**0.0030**	**0.0051**	**0.0104**	**0.0086**	1.0000	0.0857	**0.0009**	0.1583	**0.0186**	0.4699	1.0000
TUDCAvsChol	**0.0338**	**0.0119**	**0.0064**	0.0948	1.0000	**0.0482**	0.1994	0.3697	0.0786	0.1223	1.0000
TLCAvsTDCA	0.1155	0.1021	0.1714	**0.0299**	0.1038	0.4117	0.1039	0.4103	**0.0104**	0.0841	0.1705
TLCAvsCtrl	0.8202	0.1363	0.9990	0.6606	0.0828	0.3260	0.0722	0.2168	0.8917	0.4898	0.1705
TLCAvsChol	0.1632	**0.0002**	0.5606	**0.0375**	0.0828	0.1599	0.9988	0.7030	0.1313	0.0841	0.1705
TDCAvsCtrl	0.0792	**0.0333**	0.1621	**0.0226**	0.3506	0.1315	0.8135	0.6475	**0.0113**	0.0517	1.0000
TDCAvsChol	0.6694	**0.0014**	0.1001	0.1005	0.3506	0.0794	**0.0406**	0.8423	**0.0456**	1.0000	1.0000
CtrlvsChol	0.0998	**0.0001**	0.5409	**0.0184**	1.0000	0.8499	**0.0206**	0.6409	0.1126	0.0517	1.0000

The name of each row means between which two groups the comparition happened, t he name of each column means each biliary BA. *P* values lesser than 0.05 are bolded.

Table S2. *P* values with F/B ratio and four parameters of alpha diversity between groups.

	F/B	Shannon	Simpson	Chao 1 index	PD_whole_tree
TCAvsTCDCA	**0.0305**	0.2052	0.2927	0.2938	0.3306
TCAvsTUDCA	0.1167	**0.0021**	**0.0011**	0.1188	**0.0477**
TCAvsTLCA	**0.0361**	0.3245	0.4247	0.1803	0.1444
TCAvsTDCA	0.2223	**0.0343**	**0.0118**	0.1407	0.1284
TCAvsCtrl	**0.0209**	**0.0010**	**0.0003**	0.1647	0.2973
TCAvsChol	0.4911	**0.0004**	**0.0001**	0.0501	**0.0096**
TCDCAvsTUDCA	0.1127	**0.0016**	**0.0004**	**0.0278**	**0.0202**
TCDCAvsTLCA	**0.0143**	0.3796	0.2576	0.3152	0.2240
TCDCAvsTDCA	**0.0286**	**0.0123**	**0.0040**	**0.0383**	0.0600
TCDCAvsCtrl	0.3627	**0.0019**	**0.0002**	**0.0419**	0.1542
TCDCAvsChol	0.0652	**0.0006**	**0.0000**	**0.0082**	**0.0045**
TUDCAvsTLCA	0.3754	**0.0052**	**0.0047**	**0.0134**	**0.0073**
TUDCAvsTDCA	0.1480	0.2864	0.3207	0.4845	0.3645
TUDCAvsCtrl	0.1115	0.4220	0.4149	0.3427	0.0681
TUDCAvsChol	0.1159	0.3429	0.3701	0.3021	0.1750
TLCAvsTDCA	**0.0243**	**0.0284**	**0.0246**	**0.0197**	**0.0219**
TLCAvsCtrl	**0.0140**	**0.0065**	**0.0044**	**0.0207**	0.0512
TLCAvsChol	**0.0138**	**0.0024**	**0.0016**	**0.0042**	**0.0022**
TDCAvsCtrl	**0.0242**	0.3250	0.3684	0.3680	0.1898
TDCAvsChol	0.2059	0.1872	0.2193	0.2988	0.1409
CtrlvsChol	0.0518	0.2382	0.2545	0.1512	**0.0083**

The name of each row indicates which two groups were compared. *P* values lesser than 0.05 are bolded.

Table S3: PERMANOVA analysis of gut microbiota with weighted_unifrac distance

	TCA	TCDCA	TLCA	TDCA	TUDCA	Ctrl	Chol
TCA		***0.0044***	***0.0035***	***0.0005***	***0.0002***	***0.0037***	***0.0004***
TCDCA	***7.11***		*0.2803*	***0.0118***	***0.0058***	***0.0314***	***0.0009***
TLCA	***12.27***	*1.20*		*0.088*	***0.0566***	*0.1559*	**0.006**
TDCA	***39.16***	***8.46***	*2.90*		*0.7645*	*0.7381*	*0.7532*
TUDCA	***43.97***	***9.79***	***3.52***	*0.08*		*0.5933*	*0.8044*
Ctrl	***24.34***	***5.90***	*1.94*	*0.20*	*0.26*		*0.7305*
Chol	***131.00***	***16.50***	***6.48***	*0.68*	*0.59*	*0.73*	

Pseudo-F values of the PERMANOVA tests are italicized, *P* values lesser than 0.05 are bolded, and *P* values are regular font

Table S4. The gut microbiota taxa at the genus level most strongly correlated with the location of 1st and 2ed axis of the principal coordinates (PCoA) analysis (r values)

	PCoA1	PCoA2
Cetobacterium	**-0.97**	0.18
Bacteroides	**0.75**	**0.63**
Lachnospiraceae	**0.75**	**0.32**
Citrobacter	**0.51**	-0.04
Delftia	0.41	**-0.73**
Enterobacteriaceae	0.45	**-0.70**
Rhizobiales	0.27	**-0.55**

P values lesser than 0.05 are bolded

Table S5. *P* values of test for 32 bacterial taxa at genus level overall groups and between each experimental group and Ctrl group.

	All groups	TCA vs Ctrl	TCDCA vs Ctrl	TUDCA vs Ctrl	TLCA vs Ctrl	TDCA vs Ctrl	Chol vs Ctrl
Meiothermus	**0.0142**	**0.0448**	0.0903	1.0000	**0.0426**	0.1200	0.1812
Clavibacter	**0.0014**	0.0674	0.1219	0.1812	0.1527	**0.0378**	**0.0306**
Bacteroides	**0.0032**	**0.0428**	0.2737	0.1675	0.1792	0.1089	0.1791
Bacteroidaceae	**0.0105**	0.0667	0.0923	0.0801	0.1262	0.1668	0.2813
Pasteurellaceae	**0.0356**	**0.0309**	0.1352	0.0665	0.1609	0.4668	0.3414
Flavobacterium	**0.0253**	0.0698	0.1802	0.1607	0.0729	0.2787	**0.0433**
Brevibacillus	**0.0055**	1.0000	1.0000	**0.0223**	**0.0421**	0.1036	0.1294
Staphylococcus	**0.0426**	0.1150	0.0874	0.1812	0.1487	0.1671	0.1812
Lactococcus	**0.0192**	0.1262	**0.0250**	0.0915	0.0593	0.0684	0.2945
Clostridiaceae	**0.0396**	0.1175	0.1382	0.0862	**0.0408**	0.1225	0.3694
Lachnospiraceae	**0.0012**	**0.0014**	0.4130	0.1627	0.1138	0.0956	0.2431
Coprococcus	**0.0164**	**0.0002**	**0.0366**	0.3388	0.1164	0.1018	0.1367
Ruminococcaceae	**0.0094**	**0.0017**	0.1289	**0.0186**	0.1424	0.1619	**0.0400**
Erysipelotrichaceae	**0.0426**	**0.0328**	0.1108	0.0682	0.1454	0.4434	0.4756
Cetobacterium	**0.0000**	**0.0000**	**0.0215**	**0.0000**	0.0746	0.3994	0.1155
Fusobacteriaceae	**0.0008**	**0.0071**	**0.0033**	**0.0000**	**0.0077**	**0.0096**	**0.0195**
Leptotrichia	**0.0484**	**0.0222**	0.0932	**0.0014**	0.4504	0.2036	0.3367
Rhizobiales	**0.0050**	0.4033	0.1391	**0.0234**	**0.0388**	0.1185	0.0588
Rhodobacter	**0.0056**	0.2946	0.0962	0.0818	0.0630	0.2021	0.1688
Rhodospirillaceae	**0.0010**	0.1643	0.1003	0.2246	0.0792	0.2775	**0.0155**
Burkholderiales	**0.0051**	0.3557	0.0558	**0.0257**	0.2118	0.1365	0.3608
Comamonadaceae	**0.0103**	0.1398	0.1400	0.1369	**0.0272**	0.2962	0.3399
Delftia	**0.0194**	0.1751	0.0570	0.1037	**0.0199**	0.2546	0.0516
Neisseriaceae_other	**0.0095**	0.1520	0.2271	0.0680	0.1373	0.1407	0.1828
Kingella	**0.0033**	0.1332	0.0959	0.1433	0.2731	0.1468	0.1683

Aeromonadaceae	**0.0112**	0.0942	0.3983	0.2561	0.1887	0.3344	**0.0493**
Enterobacteriaceae	**0.0012**	**0.0329**	**0.0369**	**0.0009**	**0.0128**	0.1817	0.2661
Citrobacter	**0.0001**	**0.0018**	0.0826	0.0581	0.0618	0.0791	**0.0429**
Aggregatibacter	**0.0222**	**0.0114**	0.2038	**0.0000**	0.1796	0.3634	0.3560
Acinetobacter	**0.0002**	**0.0153**	0.0518	0.0685	0.0866	0.4985	**0.0003**
Pseudoalteromonadaceae	**0.0021**	**0.0383**	0.0747	**0.0005**	0.0854	0.1646	0.1027
Luteolibacter	**0.0034**	0.1205	0.1084	0.1830	0.1357	0.0786	0.0682

P values lesser than 0.05 are bolded

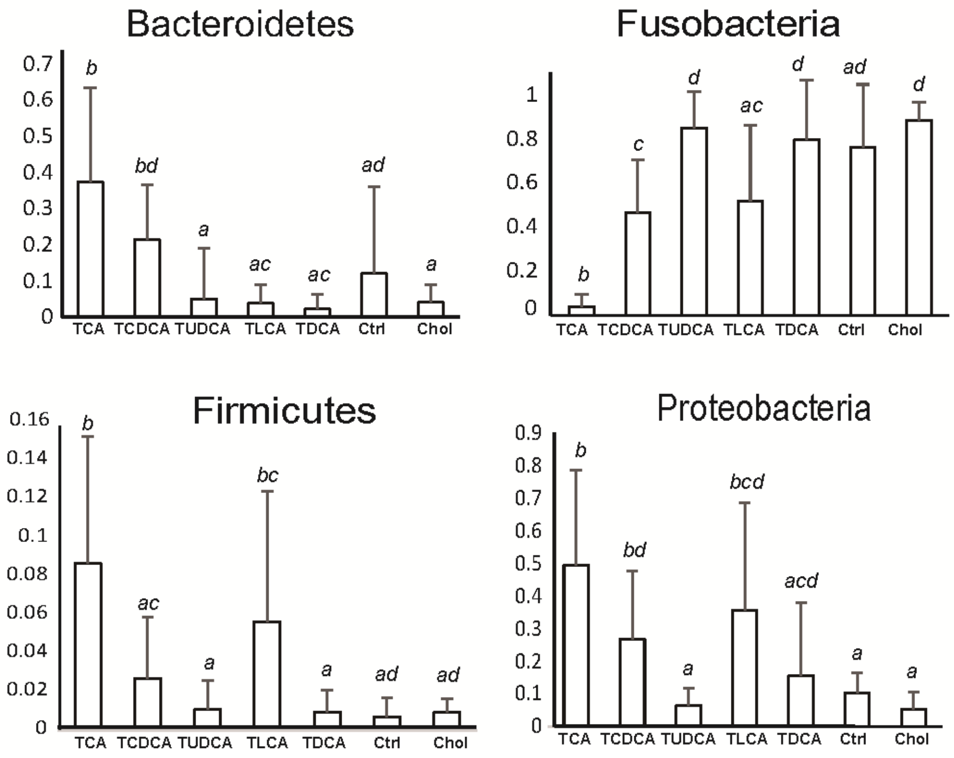

Fig. S1: Prevalence of four most abundant microbial phyla in experimental groups (x-axis). Different letters above bars indicate statistically significant (p<0.05) differences between groups. Groups not sharing the same lowercase letter are significantly different(P<0.05).

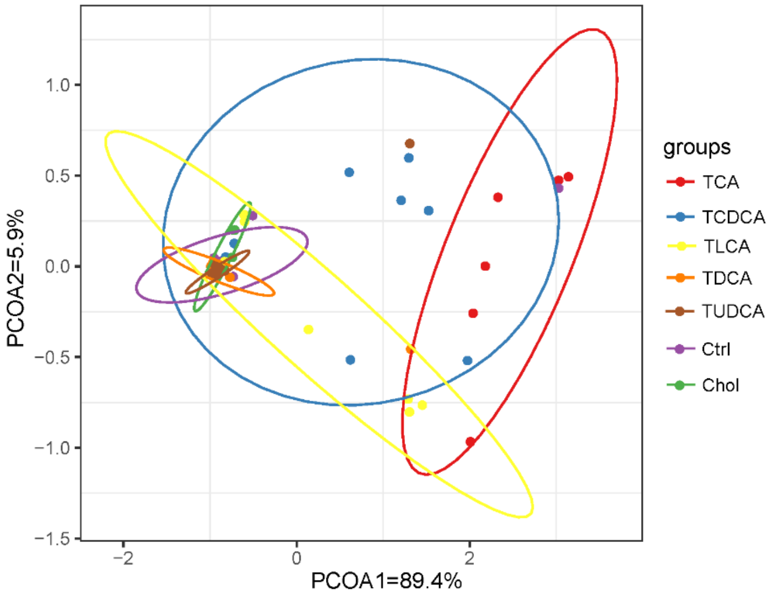

Fig. S2: Principal coordinate analysis (PCoA) performed on weighted_unifrac distance of bacterial OTUs. For the TCA fed group, n=6; for the TCDCA fed, TLCA fed, TDCA fed and Ctrl groups, n=8; for the TUDCA fed and Chol groups, n=9.
